# Thinking at the Edge: Enhancing Self-Awareness in Social Work Education

**DOI:** 10.3390/bs15030323

**Published:** 2025-03-06

**Authors:** Ofra Walter, Batel Hazan-Liran

**Affiliations:** 1 Department of Education & Social, Tel Hai Academic College, Upper Galilee 1220800, Israel; 2 Department of Education, Tel Hai Academic College, Upper Galilee 1220800, Israel

**Keywords:** Thinking at the Edge (TAE), self-awareness, metaphor, body sensing, reflective practice, social work education, professional development, embodiment, client engagement

## Abstract

Self-awareness and the ability to translate body sensing into knowledge are vital skills for social workers. This study examined the impact of a course module for master’s students in social work in Israel, incorporating Thinking at the Edge (TAE), a method for fostering self-awareness and reflection. The goal was to explore how students applied TAE to their personal development and professional practice. Twenty students participated in a modified TAE module, engaging in exercises focused on body sensing and reflection. They documented their experiences in reflection diaries, which were thematically analyzed to identify key insights about their learning process and its professional relevance. Analysis revealed the following three categories: (1) clearing the space, where students recognized and processed both negative and positive body sensations; (2) forming metaphors and patterns from the felt sense, enabling an awareness of self-patterns and behaviors; and (3) applying TAE in social work practice. Students reported increased self-awareness and acknowledged TAE’s utility in engaging with clients. The findings underscore the potential of TAE to enhance self-awareness and professional skills in social work students, offering a practical framework for integrating reflective practices into personal and client-based contexts.

## 1. Introduction

Thinking at the Edge (TAE) is a practice of self-awareness developed from Eugene Gendlin’s philosophy of the implicit and is a systematic way to think about our world and ourselves by directly referring to a felt sense (Gendlin, 1997; Morotomi, 2017). Gendlin (1991, 1997, 2004) worked on developing a complete theory of human experience for nearly half a century. The fully developed theory included a new view of the nature of the self. In moving toward his theory of human experience, Gendlin first articulated how experience functions before language, thought, feeling, and any other form of symbolization, such as concepts and theories. He then developed critical insights into radical experiential empiricism placing experience first, thus reversing the more common Newtonian ordering in which concepts organize and derive phenomena (Gendlin, 1997). Experience, for Gendlin, is something we inwardly refer to “naturally” as we go about our daily activities, often paying little attention. He extends this central notion of the natural side of human experience to include all living organisms, asserting that every living organism is in ongoing bodily interaction with a complex situation and the universe (Gendlin, 1997, 1991).

Most of Gendlin’s work on TAE has been with adults (Gendlin, 2004), but some of his associates have explored the applicability of TAE to creative thinking with students. Larrabee, for example, describes a series of six one-hour sessions she held over one semester with a class of 13-year-olds (eighth graders) (Larrabee, 2004). They practiced finding an internal, embodied “felt sense” to help them discover their “creative edge”. Students were asked to think of an area of their life that was significant but puzzling to them and see if the felt sense could give them the beginnings of a clearer picture. They were taken through a series of steps and asked to write something that captured their new understanding, and they shared this with family, friends, and teachers. According to Claxton, TAE has the potential to assist in the development of a positive learning disposition (Claxton, 2006). Much real-world learning involves the intelligent use of rich impressions rather than the simple acquisition of information.

New methods that concentrate on the body’s felt sense or the mind–body–spirit paradigm are increasingly being adopted by health professionals to promote health and healing. Such paradigms could be beneficial to the social work profession (Raheim & Lu, 2014). A few social work students have studied related modalities during graduate training (Raheim & Lu, 2014). In our study, students working toward a master’s in social work in Israel were exposed to a course that focused on body experience; one module of the course included practicing TAE to develop self-awareness and learning how to use it as a tool with clients.

### Thinking at the Edge (TAE)

The TAE process starts from a knowledge of that which a person does not yet have words to talk about. For instance, if a person is experienced in a particular field of endeavor, s/he might have a vague but persistent feeling of having something to say about that area that no one else is saying. TAE encourages the individual to develop a new use of language, an “experiential intricacy”, to express a new sensed experience, finding precise words that fit what s/he wants to say by letting them emerge from the felt sense and then checking back with the felt sense (Tanaka, 2010). Language develops in a way that frees people from being trapped by assumptions, released from the constraint of defined concepts; newly generated phrases point to aspects of experience that cannot otherwise be formulated. In other words, TAE is a method for giving expression to something we cannot explain, even if we are sure what it is like. As such, it allows us to verbalize experience (TAE Reflection, 2025).

**Figure 1:**
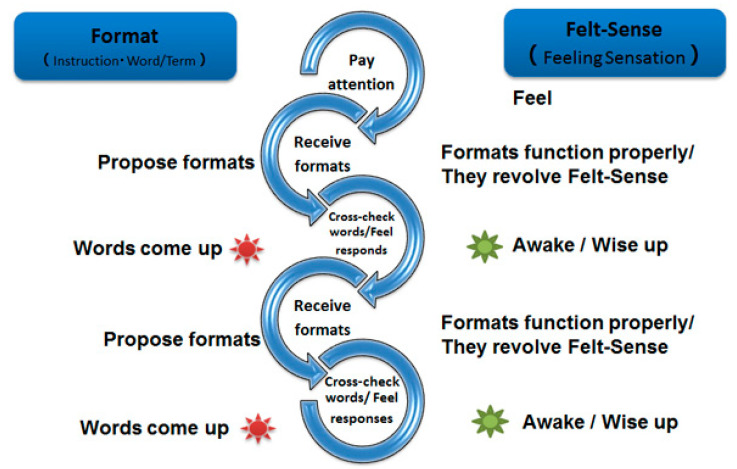
The TAE process.

TAE comprises 14 steps (see Figure 1). As Gendlin argues, “Making this method systematic is not only useful in thinking, but reveals a whole new field of rules, a new kind of logic, a new way of understanding what the powers of thinking always were, and strong additions to these powers” (Gendlin, 1997, p. 206). The 14 steps are as follows: steps 1–5, speaking from the felt sense; steps 6–8, finding patterns from facets (instances); step 9, writing freely; and steps 10–14, building theory. The process produces a word or term, which is tangible, from the felt sense (feeling sensation), which is intangible, and then expands on the word to create meaning.

The felt sense is an individual’s meaningful sense of a situation or experience. It is a daily experience, and it permeates the individual’s thoughts and behaviors. Some people may not be aware of it, but they can access it through self-reflection. The sentence is a short sentence (or phrase) that compacts the essence of a felt sense. The pattern is a short sentence extracting a general relationship (how things work) from specific experiences. It may appear in other experiences, and it helps predict and discover commonalities. The conceptual system is a short sentence expressing the concepts of values by connecting keywords. It responds coherently according to the situation and works as a behavioral principle beyond a time and place.

By focusing on and recalling the felt sense of self, we can navigate our thoughts and behaviors. Thus, some researchers have examined the use of TAE in a therapeutic context. Japanese therapist Yoshihiko Morotomi studied it as a method of reflection on clinical cases and as a method of theory building for person-centered and experiential psychotherapists (Morotomi, 2017). Tanaka described treating a client using TAE and summarized the process as sensing, finding words, identifying the new concept, and crossing into multidimensional space (Tanaka, 2010). We drew on this process in our research with master’s students in social work (MSW). We argued that incorporating TAE in social work education would enhance students’ self-awareness, foster emotional resilience, and deepen reflective practice. By engaging in TAE, social work students could identify hidden emotions and behavioral patterns, improving their capacity for empathetic client engagement and holistic decision making (Morotomi, 2017), strengthening their professional growth, and supporting transformative social work practice (Gendlin, 1991, 1997, 2004).

Social work involves complex emotional demands; social workers must manage their own emotions and promote the wellbeing of others (Barlow & Hall, 2007). They can use TAE to enhance their self-care strategies, while improving their social work skills with their clients (Napoli & Bonifas, 2011). We argued an ideal opportunity to teach TAE would be in advanced social work courses, and we focused on MSW students in Israel for two reasons. First, the Israeli curriculum is stressful in and of itself. Second, most students are managing professional and personal lives; in Israel, professional social work practice generally begins after the completion of a three-year undergraduate degree, so MSW students generally have practical experience in the field. Many continue to work during their graduate studies. TAE may help them handle this difficult balancing act, as it develops concentration, deepens understanding and insight, and cultivates awareness (McGarrigle & Walsh, 2011; Wang, 2013). However, a paradigm that supports the use of body sense as a framework to promote both practitioners’ health and clients’ health is seldom incorporated into MSW studies. We examined how the MSW students in our sample experienced the integration of body awareness into their social work practice and explored the benefits and challenges of incorporating TAE into the MSW curriculum.

## 2. Materials and Methods

### 2.1. The Course Model

Based on the literature, we designed a course as an experiential learning environment for MSW students, focusing on the role of the body in the context of clients and therapists. Although incorporated into their studies, the course was based on their practical experience in the field. We took an intervention approach, viewing learning as an active process, engaging the learner (in this case, the MSW student) in constructing knowledge and adjusting the cognitive framework to accommodate new information related to current and future professional practice.

Social workers are called upon to treat people experiencing stressful life events. At times, words may be insufficient; nonverbal methods may be more suitable and less threatening. For example, children’s exposure to violence and traumatic events may lead to malfunctioning in the Broca, the area of the brain controlling speech (Crenshaw & Hardy, 2007; Walter & Golan-Shenaar, 2018, 2019). Social work requires the ability to perceive, understand, experience, and respond to the emotional state and ideas of another person (Trevithick, 2008), and the course was meant to foster the ability of social workers to sense the body and create an account of the body sense to realize what patterns and behaviors were leading them and their clients (Madison, 2014). It focused on sharing, learning, and reflecting on body sensing, perceiving, making sensing into knowledge, and understanding patterns and behavior. Thus, in this intervention course, students explored strategies whereby they became aware of their physiological arousal, behavior, and patterns, realized the effects on their emotional understanding and performance of self and others, and developed strategies to exercise control/regulation over physiological states (Gibbs, 2002).

The course module comprised 13 sessions of 90 min Zoom sessions, since it was taught during the COVID-19 pandemic. Sessions focusing on body gestures had to be modified for Zoom by using the camera video. The first seven sessions focused on gaining awareness of the body through different approaches, such as body gestures and play activities engaging students on several dimensions—verbal and nonverbal. They included the learning modalities of imagery, movement, and roleplay. The eighth to tenth sessions focused on TAE, the topic of this paper, and the three last sessions featured approaches to emotional abilities.

In the TAE section of the course, students were encouraged to explore self-awareness and interpersonal awareness and were taught the basic steps to translate this awareness into behavioral changes and changes in their feelings and belief systems. For students to feel safe experimenting, a classroom group dynamic using Zoom rooms enabled them to practice and experience, while fostering support, validation, and acceptance of self and others in the group. The students recorded their TAE session experiences verbatim in diaries to capture the details of self-awareness and shared their experiences with the group during the Zoom sessions.

In the first session on TAE, students were exposed to the theory. Then, the lecturer demonstrated the process to one of the students; the students were given written guides to the TAE process and asked to practice with a partner. They were divided into Zoom rooms; one of the partners guided, and the other experienced. At the end of this session, they shared their experiences with the group. The process followed these key steps:

1. Focusing on an experience: students were asked to choose an instance that stood out as different from usual experiences and to pay attention to its qualities and the felt sense it carried.
2. Enriching details: students were asked to expand upon the sensations, emotions, and elements present in the experience and to move beyond common words to describe it in more precise and nuanced ways.
3. Identifying patterns: students were asked to notice emerging structures within the experience and to begin formulating connections transcending personal interpretation.
4. Creating patterns with words: students were asked to express insights in new formulations, allowing a fresh understanding to surface, and to experiment with wording to find expressions capturing the deeper meaning.
5. Exploring connections between memories: students were asked to compare a present experience with a past one, particularly a childhood memory, to reveal hidden self-patterns.
6. Refining understanding: finally, students were asked to adjust their statements and explore alternative expressions until they felt accurate and meaningful.

Throughout the process, they were encouraged to ask guiding questions, such as “What does this reveal about itself rather than about me?” or “What deeper structure is emerging?” to help refine insights. Afterward, students recorded their reflections in personal diaries.

In the second session, students watched a Gendlin video on TAE and revisited the steps practiced in the first session. They, then, engaged in another round of paired practice in Zoom breakout rooms, followed by group reflections and diary entries.

The third session focused on exploring childhood memories to uncover self-patterns influencing present behaviors. The exercise involved the following:

1. Selecting a present memory (A): students were asked to identify the memory’s felt sense and mark a word.
2. Selecting a childhood memory (B): students were asked to identify the memory’s felt sense and mark a word.
3. Connecting A and B: students were asked to formulate a sentence that captured the relationship between the two memories (e.g., “A is a type of B”).
4. Refining the statement: students were asked to adjust wording until it accurately reflected the underlying connection.

The guiding partner facilitated the process with reflective questions, such as “How does A relate to B?” or “What hidden connections exist between A and B?”

By the end of the third session, students had gained deeper insight into how past experiences shape current perspectives. They documented their reflections in diaries, reinforcing the learning process.

### 2.2. Measurements and Methods

We used a qualitative approach and a grounded theory approach to assess the potential for TAE to be integrated into social work practice. To determine the potential value of TAE, we thematically analyzed the reflective diaries participants completed in the course. The reflective diaries were maintained by all students but were not part of their final mark. In the TAE module, students documented their partner’s TAE practice experience and vice versa. They then reflected on their personal experiences with the process. At the end of the sessions, students submitted their reflective diaries anonymously.

To analyze the data, we employed a grounded theory approach, following Corbin and Strauss’s methodology (Corbin & Strauss, 2008). In an initial coding phase, two independent coders systematically reviewed the diaries and identified recurring themes and concepts emerging from the data. They used axial coding to establish relationships between themes, allowing for a deeper understanding of the cognitive, emotional, and practical engagement of students with the TAE process. Finally, selective coding was applied to refine the core categories encapsulating the essence of students’ experiences.

To enhance the rigor of our analysis, we used constant comparison techniques, in which data segments were continually re-examined to refine thematic categories and ensure internal consistency. Discrepancies between coders were resolved through discussion until consensus was reached. The final analysis resulted in the identification of three overarching categories and several subcategories. The categories were (1) clearing the space, (2) forming metaphors and patterns from the felt sense, and (3) applying TAE in social work practice.

### 2.3. Participants

The participants were 20 MSW (body–mind psychotherapy) students in an academic college enrolled in a course in their final year of study during the COVID-19 pandemic (19 females and one male, ages 24–35). They lived in varied places in Israel. The course was conducted on Zoom. In total, 3 participants were native Arabic speakers; 17 were native Hebrew speakers (note: Hebrew is the official language for all courses at the college). The college’s mission is to produce dedicated, open-minded professionals in the direct practice of social work with individuals, families, groups, and communities in urban and rural environments (Mosek & Ben-Oz, 2011). The course was one of many mandatory courses in the curriculum; it focused on the body’s role in the environment between social work and client, with an emphasis on body sensation.

All participants were notified about the research procedure and signed consent forms before the first class meeting. The research was awarded internal approval by the Ethics Committee of the college where the research took place. The first author conducted the course; she has specialized in using nonverbal methods to develop emotional awareness in various fields of education, including in the social work field.

## 3. Results

The analysis showed how the basic steps of TAE practice impacted students’ understanding of the bodily invitation process and their ability to listen to their bodies and understand their chosen instance, ultimately creating a sense of their self-patterns and behaviors. It also revealed their sense of TAE’s value in their interactions with clients. We identified the following three main categories and several subcategories: (1) clearing the space: (a) awareness of body negative sensing, (b) awareness of body self-connection, and (c) breathing as clearing space and grounding; (2) forming metaphors and patterns from the felt sense: (a) metaphor, (b) paradox, and (c) crossing; and (3) using TAE in the MSW profession.

### 3.1. Clearing the Space

Clearing the space was the first category; it had three subcategories.

#### 3.1.1. Awareness of Negative Body Sensing

In the first stage of TAE practice, students were instructed to clear a space by focusing on breathing. They were asked to distinguish between sounds outside the body and directing attention inward. The diary reflections revealed varied experiences. Some participants described a sense of detachment or resistance, while others struggled with emotional and cognitive overload. For some, resistance manifested as skepticism or discomfort, often linked to an underlying reluctance to confront internal content. For example, D reflected on feeling detached and cynical, despite recognizing a simultaneous desire for growth and self-exploration. She questioned whether her resistance stemmed from a genuine disconnection or an unconscious avoidance of emotions she did not want to face. Similarly, N described frustration and difficulty achieving a sense of calm, reporting feelings of anger, nervousness, and mental flooding when attempting to focus inward.

These responses suggest clearing the space was a pivotal moment in the TAE process, where students confronted barriers to self-awareness. While meant to foster presence, it can initially heighten discomfort before leading to deeper insight. Rather than being an obstacle, however, resistance signals a meaningful point of friction that, when explored, promotes emotional clarity. The experience of negative body sensing further revealed the tension between control and surrender in reflective practices. Some students struggled to let go of cognitive control, resulting in emotional turbulence. This highlights the need for structured guidance to help participants navigate somatic awareness without becoming overwhelmed.

#### 3.1.2. Awareness of Body Self-Connection

For some students, the invitation to clear the space aroused a desire to connect body and mind to the self. N noted:

My need for a connection between body and mind has been growing stronger and opportunities to explore it are constantly appearing. I was not familiar with the focusing tool, but I knew that it can help me extract information from myself that I need very much but that I cannot access due to emotional barriers. Today, I understand that those feelings that arise in me are a significant and inseparable tool from the rest of my therapeutic tools in the clinic.

Thanks to her professional experience, N was able to bridge theory and practice, cultivating a curiosity about this connection and a desire to continue exploring it. As she put it, “It feels like I’ve ’come home’. I’m not sure where I went, or maybe I didn’t go anywhere at all, but I felt distant, and it feels so good to return”.

#### 3.1.3. Breathing as Clearing Space and Grounding

The invitation to clear the space by focusing on breathing allowed repetition and a focus on the here and now, thus permitting a transition from a state of wakefulness and control of thoughts to ingathering and attentiveness. Some students initially found focusing on breathing to clear a space was difficult. For example, L encountered resistance and explained working through it as a physical process:

Breathing for me is inner work. Over time, as I practiced more as a focuser and in focusing and listened to the lecturer’s instructions, I truly ‘cleared space’ in my mind. I was able to feel more the breaths in my body, to breathe deeply, imagine shapes, colors. During the breathing phase I experienced a lot of physical sensations along with thoughts that prevented me from concentrating, but when I was asked to ground myself on the soles of my feet, I achieved a different sense of security.

S mentioned this as well, along with her initial difficulty: “I felt that I too as a focuser needed this transition that would help me let go, release irrelevant thoughts, and connect with myself and focus. I felt a little stressed, because this was my first experience focusing”. Y commented, “I felt how I allow myself to stop and turn my attention inward, to quiet the train of thought and separate the voices outside from those inside”. The feeling was one of release, relaxation, and calm, as noted by H and SI, respectively:

I suddenly began to notice that my body is alive, noticeable, vague feelings that come and go all the time, and if I put my attention openly to the existing feelings.

To feel where I sense it in my body and also to find out what I want to do with it. It gave a place to emotions that had received no attention from me.

In summary, clearing the space through a focus on breathing, although difficult, allowed the cessation of thoughts and consciousness and permitted a greater awareness of body sensations; this, in turn, allowed a deeper understanding, not just of sensations but also of oneself. As L said, “Overwhelmed with feelings of uncertainty and anxiety that are manifested in physical manifestations in the digestive system, and by awareness of these feelings and proper breathing, anxiety can be reduced and emotional work done”.

### 3.2. Forming Metaphors and Patterns from the Felt Sense

In the stage after clearing the space, the focuser chose an instance and directed his/her focus to the sensation, identifying what the feeling was. The guide (the student partner) asked the focuser to try to place a name on the feeling in different ways (metaphor, word, etc.). The focuser named the word or wording that arose and examined it vis-à-vis the sensation. This process occurred in two parts, one related to a current memory and one to a childhood memory. The wording that emerged from the sensations in both instances pointed to a second subcategory, paradox, and this was followed by a third subcategory, a process of crossing into understanding.

#### 3.2.1. Metaphor

The clearing of a space and the statement of the instance allowed students to be in touch with the feeling and check how the word, phrase, or sentence they derived described the sensation. Some found a metaphor or word that was authentically different and unique for each instance. For example, M said, “Anxieties and fears came over me like a heavy stone lying on my chest. There was a barrier, distress like a weight and the tears came very easily”. N’s imagery was interesting:

As I focused on these feelings the emotion changed to sadness I felt as a vague discomfort in my chest and throat. From these feelings came the notion of ‘peace in the sadness of life’, which I felt as a small pillar in the center of my stomach.

S said:

The image that came to me, which describes the experience I had in the experience, is ‘going on a shared journey’. I found myself thinking with the listener, writing down everything he said, feeling grateful and privileged for the listener’s ability and willingness to dedicate himself to this journey.

As the diary reflections indicated, this stage was a significant step in the discovery and understanding of emotional patterns. The metaphors created by the students did more than just describe their emotions; they served as bridges to deeper self-awareness. By articulating their experiences through metaphors, the students not only externalized their inner worlds but also began to understand how their emotions could be navigated and transformed. Each metaphor was a key to unlocking new dimensions of emotional experience, guiding students toward a more integrated sense of self. In this way, the use of metaphor served as both a tool for expression and a path towards healing.

#### 3.2.2. Paradox

The concept of paradox played a central role in the students’ self-discovery and understanding of their behavioral patterns through focusing. In this context, the paradox emerged from the students’ realization that seemingly unrelated instances, one from the present moment and another from childhood, had striking similarities in emotional experience, bodily responses, and behavioral patterns. This process highlighted how these past and present experiences, despite the passage of time, were interconnected in ways that revealed recurring patterns lasting into adulthood, a phenomenon called “paradox” in TAE.

K’s experience illustrated this paradox. She described a contemporary conflict at work, where she felt threatened and defensive, experiencing physical sensations of pressure in her chest and shoulders, as well as emotional responses of helplessness and shame. When she recalled a childhood memory of being influenced by peers to hurt another child, she again encountered shame and physical sensations—heat in her throat and a feeling of discomfort. Despite the different contexts—one in adulthood and the other in childhood—K’s bodily and emotional responses were remarkably similar. Her metaphor “you have to be on your guard” arose from both experiences, indicating a long-standing pattern of alertness and avoidance stretching across the years. This paradox challenges the common assumption that childhood and adulthood are distinct phases of life, separate in experience and influence. In reality, the emotional responses and behaviors developed in childhood often remain active and unresolved into adulthood, where they continue to shape how individuals respond to stress and conflict.

N’s story revealed how her early childhood experiences, when language was not yet developed, had a profound effect on her. Her memory of walking towards one cousin only to be picked up by another, causing frustration, led to a bodily sensation of sadness in her stomach. As she reflected on this sensation, a metaphor emerged: “restless rhythm”. This metaphor evolved into a more complex understanding of the interconnectedness of the body and emotions as the imagery shifted, first representing an arc and later spreading throughout her body. The child’s experience was vague and nonverbal, but it held within it the seeds of a pattern that became clearer much later. Despite the lack of language in childhood, the physical sensations and emotional content of that early experience were carried forward into adulthood, ultimately leading to a moment of clarity and liberation in N’s reflection. This suggests that the body and emotions retain memories and patterns from childhood, even when conscious recollection may be fragmented or unclear.

The paradox also arises from the notion that insights into one’s behavior can emerge not through logical reasoning but through the identification of bodily sensations, metaphors, and fragmented memories. Y and H both expressed surprise at how metaphors and bodily knowledge allowed them to discover deep insights about themselves. Y, as a listener, was amazed by the focusers’ ability to connect bodily sensations with stories, dreams, and childhood memories to form new understanding. Similarly, H found joy in discovering how deeply ingrained bodily knowledge could offer new clarity and understanding of herself. This demonstrates how the body can serve as a repository of both past and present experiences, offering insight into recurring patterns of behavior that might otherwise remain hidden.

Ultimately, the paradox lies in the realization that past and present behaviors, though they may seem different on the surface, are inextricably linked through emotional and bodily responses. These patterns persist across time, shaping individuals’ reactions to similar situations. The process of focusing on these bodily experiences allowed the students to access and understand the deeper structure of these repeating patterns, offering the possibility of healing and growth by breaking the cycle.

#### 3.2.3. Crossing

The concept of crossing in the TAE process represents a pivotal phase where individuals move beyond simply identifying paradoxes in their experiences and behaviors and begin to integrate these insights into a new level of personal understanding and transformation. It is a phase that bridges the past and present, enabling individuals to reframe their emotional and cognitive patterns, ultimately fostering growth and change. At the heart of the crossing phase is the realization that the paradoxes discovered in the earlier stage—such as the connection between childhood and adult patterns—are not just intellectual concepts but deeply embodied experiences that shape how individuals respond to the world. This realization, as seen in Y’s reflection, underscores the importance of crossing over from old patterns to new ways of engaging with oneself and others. Y recognized a pattern of helplessness that stemmed from childhood and continued to be triggered in adulthood when she felt insecure. By focusing on the present and identifying these long-standing emotional reactions, she was able to break free from her habitual ways of responding. This insight led her to a profound understanding of how detaching from the familiar—stepping outside her usual ways of thinking—allowed her to hear her inner voice more clearly. The crossing was not just the awareness of the pattern but also the movement toward the courage to act differently, creating space for new learning and action in the future.

N’s experience similarly illustrated how crossing involves transcending the intellectual and habitual ways of thinking to access deeper, more authentic emotional experiences. N spoke about how focusing helped her detach from intellect, which she described as a barrier to feeling her authentic emotions. The process forced her to deal directly with the feelings in her body, confronting the deeper, often hidden parts of herself. This stripping away of intellectual defenses allowed a clearer direction of change. In this context, crossing was a transformative act; the shift from intellectualizing feelings to fully experiencing them, allowing the body and emotional truth to guide the way forward. N shed old layers of intellectualization and moved into a deeper, more embodied understanding of herself.

K’s reflection shows how crossing works as a means of linking past and present patterns to form a coherent understanding of oneself. By focusing on the bodily sensations tied to her concepts of “alertness” and “embarrassment”, she recognized these two feelings were deeply connected, each reinforcing the other. Through this realization, K was able to understand how weakness and helplessness had shaped her responses both in her childhood memory and in her current life. This connection between the past (childhood) and the present (adult) was not merely a cognitive understanding but a felt experience that resonated in her body. K’s insight into the interconnection of these sensations and emotions represented a crossing of temporal boundaries, where past and present no longer existed in isolation but came together to create a more holistic understanding of her emotional responses.

In all these cases, the crossing phase allowed significant insights by shifting away from intellectual thinking and creating space for embodied awareness. Through this embodied understanding, participants were able to confront long-standing patterns of behavior and emotion, whether from childhood or adulthood, and begin to reshape how they engaged with themselves and the world. This process of crossing from automatic responses to mindful awareness led to a deeper connection with the self and greater clarity about the necessary changes to make. Ultimately, the crossing phase marked a profound transformation. It involved moving from intellectual understanding to embodied knowing, from passive observation to active change. The practice of focusing and engaging with the body allowed participants to cross over from old, automatic patterns to new forms of self-awareness and action, paving the way for personal growth and healing. The crossing was not just a theoretical understanding of patterns but an experiential one that redefined how they engaged with their own emotional and psychological worlds.

### 3.3. TAE Uses for the Social Work Profession

The students saw the TAE approach as highly valuable for their work in social services, emphasizing its potential to foster deeper connections between the body, mind, and the therapeutic process. By utilizing body sensations as a source of knowledge, TAE offered a unique tool that could enhance their professional practice.

K highlighted the therapeutic significance of focusing, noting how it allowed her to connect with her clients more authentically:

I can be more targeted to my patients and in the proper way. As a therapist, there is a critical meaning to this process of observation, from the breathing to the body sensations that the words helped to produce. The more I practice focusing and tuning into my body, the more I can be in sync with my patients in a real-time, authentic way.

K thought the process of focusing, whereby she paid attention to body sensations, enabled her to better empathize with and respond to her clients’ emotional needs.

D and N saw focusing as a catalyst for growth, connecting the body and mind, “Clearly, we both began with an understanding of the importance of connecting the body and mind in our lives and perceptions. The focusing tool served as a stimulus for growth”. Both D and N emphasized the optimism inherent in the process, believing everyone holds the capacity for growth within, even if it is not immediately accessible. As N put it, “Everything happens with a cause that enables growth and development, and that is concealed within us”. This belief in the inherent healing potential aligns with the values of social work, where practitioners seek to empower clients to uncover and process their sources of strength.

For K, focusing also opened a path for a more personalized therapeutic language:

The technique is simple, allowing access to very deep and vulnerable content, gently, without compromising defense mechanisms. The body knows how and where to aim. It can be used in many situations to allow the individual to explore their experience with the intimacy that the body wisely navigates.

This perspective reflects the uniqueness of TAE for social work, where practitioners can guide clients through deep emotional exploration using a nonverbal, embodied approach. This method can foster a safe space for clients to engage with difficult emotions, bypassing traditional defense mechanisms.

S echoed the sentiment, noting how focusing helped her connect more fully with herself, “My body and mind are very much connected. My body language often says what is difficult for me to express verbally. Sometimes I transmit a rigid and unpleasant energy to my environment and thus convey the message”. This underscores the role of body awareness in understanding and addressing the unspoken aspects of communication, which is critical in social work contexts, where nonverbal cues often reveal hidden emotional struggles.

Y, M, and D pointed to the value of Thinking at the Edge—a key aspect of TAE that allows individuals to access “felt knowledge” and “hidden knowledge” that often cannot be expressed through words alone. Y shared, “Focusing connects us to body consciousness naturally and intuitively. It contrasts with traditional therapies that rely only on verbal discourse. It brings about change in understanding our inner world, challenges, and attitudes, almost like using a magic wand to unlock new insight”. The ability to access unspoken truths offers social workers a powerful tool for understanding clients’ deeper struggles, even when those struggles cannot easily be articulated.

M also emphasized the accessibility and utility of focusing on everyday life: “Focusing allows for the recognition of felt senses that haven’t yet been addressed. These feelings won’t emerge through the mind or emotions but only through the body. Focusing reveals patterns and generates change by acknowledging difficult feelings that might otherwise remain hidden”. The versatility of TAE as an easily adaptable tool in social work practice, applicable in any setting, makes it a valuable addition to a social worker’s toolkit.

D’s reflection encapsulated the transformative power of focusing: “I find myself enveloped in the feeling that I have returned home… The starting point through which I experience the world is that there is a direct, fascinating, and obvious connection between body and mind”. This sense of “returning home” speaks to the profound reconnection with one’s authentic self, an essential aspect of healing and growth in social work.

The partner guides also recognized the therapeutic value of TAE. For example, S observed the progress of the focuser, saying, “I felt good. The focuser managed to conceive new concepts, and I felt I had dedicated myself to being present with him in the here and now, without the worries of the day”. This highlights the dual benefit of TAE; not only does it facilitate personal insight for the focuser (client), but it also fosters mindfulness and presence for the guide (social worker), creating a therapeutic dynamic where both parties engage deeply with the process.

In conclusion, the diaries suggested that TAE offers a unique and effective tool for social workers to engage with clients on a deeper, more embodied level. By facilitating connections between body sensations, emotional awareness, and verbal expression, TAE encourages both personal growth and professional development. It helps clients uncover hidden emotions and patterns, fosters authentic self-expression, and promotes healing and change. Beyond personal insight, TAE provides social workers with an innovative, adaptable method to enhance their practice, making it a valuable tool for those seeking to foster growth and healing in their clients.

## 4. Discussion

Although recent work supports the use of mind–body approaches to promote the health and healing of social workers and their clients, only a few social workers have had the opportunity to study this paradigm and related modalities during graduate training (Raheim & Lu, 2014). This study examined the value of incorporating a modified version of TAE into a course for MSW students. Specifically, it sought to understand how MSW students experience the integration of body awareness into their social work practice, as well as the perceived benefits and challenges of incorporating TAE into the MSW curriculum.

TAE has rarely been integrated into training programs, even though it has the potential to enhance students’ critical thinking, create meaningful learning experiences, and contribute to their professional identities and their wellbeing, as well as that of their clients (Barlow & Hall, 2007). The results of the diary analysis showed that MSW students found TAE practice added both professional and personal value.

Even though it was difficult, students indicated the benefit and importance of breathing as a tool. By focusing on the here and now through breathing, they could clear a space between thoughts or clear their thoughts entirely, enabling body sensations to arise. Some students reported a positive connection to the body; they were immediately connected to the mind and invited to an inner search. Several had a negative reaction to concentrating on breathing, but this too invited them to an inner exploration, in this case, searching for the reasons for the negative approach. In both cases, they recognized emotions relating to body sensation.

In the next step, students began to experience and express an instance. Tanaka suggests that TAE encourages language experiential intricacy, wherein a clear space enables the connection of body sensation and mind (Tanaka, 2010). Students were asked to sense the instance, first of a current memory and then of a childhood memory, and to assign a word or metaphor to each instance. Finally, they explored the links (termed a paradox) between instances. Otherwise stated, they cross-referenced the past and present, and in this way, they identified personal patterns. Tanaka argues that the TAE process culminates in illumination, as the practitioner becomes aware of the multidimensional (Tanaka, 2010). In fact, the students were surprised by the insights they had in a short time. They recognized patterns and behaviors from childhood that continued well into adulthood.

The embodied cognition approach argues that cognition can be rooted in motor behavior (Kemmerer et al., 2013). TAE uses breathing in the sensing part of the process, and students consistently mentioned physicality. However, once the technique was mastered, TAE enabled an “experiential intricacy”, wherein MSW participants let words emerge from the felt sense and made meanings from them (Tanaka, 2010).

The students thought the approach could be useful in their professional field. Connecting body sensations with verbal expression was particularly useful, as they could apply this awareness to their interactions with clients. They recognized the potential for TAE to enrich their social work practice by fostering deeper emotional and cognitive connections with clients, while simultaneously supporting their wellbeing. As both focusers and guides, students found that TAE enhanced their ability to tune into their own and their clients’ felt senses, promoting a more empathetic and mindful approach to social work practice.

The findings suggest integrating TAE into the MSW curriculum can provide significant personal and professional benefits for students. It offers a powerful tool for self-awareness and emotional regulation, which is essential for social workers in managing the complex emotional demands of their profession. Despite the challenges students faced in mastering the technique, the insights gained from their experience with TAE reflect its potential to not only improve self-care practices but also to deepen practitioners’ understanding of themselves and their clients. Further research into the integration of TAE into social work curricula is recommended, as it could offer a new framework for developing more holistic, compassionate, and effective social work professionals.

### Limitations and Future Research

This study has several limitations that should be considered when interpreting the findings. First, it focused solely on the perceptions of a single group of MSW students in Israel, limiting the generalizability of the results. The absence of a control group makes it difficult to determine whether the observed effects were specifically attributable to TAE or *may* be linked to other factors, such as individual differences or the reflective nature of social work education in general. Future research should incorporate a comparison group enrolled in a course without TAE to better assess its unique impact. Future research should also explore how TAE can be adapted for different cultural contexts, diverse social work populations, and varying levels of experience, ensuring its broader applicability and effectiveness. Expanding the study to include practicing social workers could provide further insight into how TAE supports professional resilience, decision making, and client engagement in applied settings.

Second, the study was conducted during the COVID-19 pandemic, requiring online learning via Zoom instead of in-person interactions. Given that TAE emphasizes body awareness and embodied reflection, the remote learning format may have influenced students’ engagement with the practice. Future studies should examine how face-to-face instruction influences the effectiveness of TAE compared to virtual settings.

Finally, data collection was limited to the duration of the course, preventing an understanding of the long-term impact of TAE on students’ professional and personal development. To address this gap, longitudinal studies are needed to track participants beyond the course and assess how their self-awareness and reflective skills evolve over time, particularly in real-world social work practice.

## 5. Conclusions

Business, medicine, and society as a whole are adopting experiential processes. One such process, TAE, is a shared experience of the listener/guide and the focuser in connecting to the felt sense in an instance in the here and now and in an instance from early childhood memory. When TAE is used in therapy, the process invites both the client/focuser and the therapist/listener to be attentive to the felt sense. The therapist focuses on the client’s experience but also on how s/he experiences the client and the therapeutic relationship, making TAE valuable to clients and practitioners alike. Our findings suggest that using TAE as an additional tool in social work may empower both practitioners and clients to deepen their awareness, uncover hidden emotions and patterns, foster authentic self-expression, and promote healing and transformation.

## Data Availability

Data are available upon direct request to the authors.
